# Three Years After COVID-19 Vaccination, Anti-Spike SARS-CoV-2 Antibody Concentration Decreases and Is Accompanied by Increasing Anti-Nucleocapsid Seropositivity

**DOI:** 10.3390/v17111443

**Published:** 2025-10-29

**Authors:** Tomasz Anyszek, Jakub Swadźba, Andrzej Panek, Emilia Martin

**Affiliations:** 1 Medical Faculty, Andrzej Frycz Modrzewski Krakow University, 30-705 Krakow, Poland; 2 Medical Department Diagnostyka S.A., 31-864 Krakow, Poland

**Keywords:** SARS-CoV-2 IgG, COVID-19 vaccine, immune response, spike antibody, nucleocapsid antibody, humoral immunity

## Abstract

Background/Objectives: The anti-spike (S) SARS-CoV-2 antibodies confer neutralizing properties and their concentration may be related to COVID-19 protection. Anti-nucleocapsid (N) SARS-CoV-2 antibodies in mRNA COVID-19 vaccine recipients indicate infection. The aim of this study was to analyze the anti-S and anti-N titers 3 years after COVID-19 vaccination. Methods: Ninety-nine vaccinated healthcare workers provided blood samples in 2024 and filled out questionnaires about their COVID-19 history and boosters acceptance. Anti-spike and anti-nucleocapsid IgG were assessed with commercially available immunoassays, DiaSorin’s SARS-CoV-2 TrimericS IgG and Abbott’s SARS-CoV-2 IgG, respectively. Results: Three years after the primary COVID-19 vaccination, the anti-S SARS-CoV-2 antibody concentration was still high. However, it dropped in comparison to the data obtained a year before (3600 vs. 2040 BAU/mL), possibly due to the lack of boosters. In contrast, the percentage of anti-N seropositive individuals grew from 34% two years after vaccination to 40.4% after three years. Subjects with SARS-CoV-2 infection within a year prior to the antibody measurements had statistically significantly higher median anti-S concentrations than those with tentatively no contact with SARS-CoV-2 (2940 vs. 1930 BAU/mL). Conclusions: Overall, our data indicates that although the booster vaccinations’ acceptance decreases, the circulating SARS-CoV-2 stimulates humoral immunity, resulting in high anti-S antibody concentrations even three years after the vaccination.

## 1. Introduction

When the first case of pneumonia of unknown etiology was detected in December 2019 in Wuhan City, China, very few people expected that the severe acute respiratory syndrome coronavirus 2 (SARS-CoV-2) would take the lives of seven million people worldwide by the end of 2023 [1].

The swift implementation of coronavirus disease 2019 (COVID-19) vaccines at the beginning of 2021 resulted in 73% of the total population of the European Union (EU) being fully vaccinated at the beginning of October 2023 [2]. This visibly reduced the number of confirmed COVID-19 deaths in the EU, from 1184 per million people in 2021 to 24 per million in 2024 [3].

Humoral immunity is one of the mechanisms induced by vaccination and can be easily assessed by anti-SARS-CoV-2 antibody testing. Therefore, it has been extensively used for both vaccine efficacy trials [4] and in post-marketing research on the response to vaccination. At the pre-clinical stage, immunogenicity of mRNA-based vaccines was proven mostly by neutralization tests, which are not feasible for conducting in routine medical laboratories. Then it was shown that the neutralizing function of the antibodies is mainly associated with the anti-SARS-CoV-2-recognizing spike (S) protein of the virus [5,6]. This led to the wide implementation of immunoassays measuring the concentration of these antibodies in clinical laboratories.

No formal recommendation on SARS-CoV-2 antibody testing after vaccination has been made thus far. However, research data showed that it can be useful in identifying vaccine non-responders, detecting decreases in the antibody titers and estimation of the level of protection against symptomatic COVID-19. Simultaneous measurement of antibodies recognizing distinct SARS-CoV-2 antigens—the aforementioned spike (S) and nucleocapsid (N)—allows for a more in-depth assessment of the humoral immunity to SARS-CoV-2, as it differentiates the source of immunity [7]. Anti-S antibodies are induced by vaccination as well as following natural infection. On the contrary, anti-N antibodies are not induced in COVID-19 mRNA vaccinees, since these vaccines only carry spike protein mRNA, and therefore indicate natural infection.

Our observational study follows a group of healthcare workers (HCWs) who received the Pfizer–BioNTech Comirnaty vaccine. This work presents data on anti-spike and anti-nucleocapsid SARS-CoV-2 antibody concentrations at a timepoint 3 years after the primary vaccination, attempting to provide insights into the humoral immunity, stemming from inoculation but also affected by booster dosing and COVID-19 incidence over the preceding year. This type of real-world data may inform future vaccination strategies.

## 2. Materials and Methods

### 2.1. Participants and Study Design

Ninety-nine Polish healthcare workers vaccinated against COVID-19 with the Pfizer–BioNTech Comirnaty vaccine between January and March of 2021 were recruited for this study. Since then, serum anti-SARS-CoV-2 antibody (Ab) levels and COVID-19 incidence have been monitored. The details of the previous findings have been published elsewhere [8,9,10,11], and this work describes the observations at a median of 3.25 years (3–3.50) after the primary vaccination, hereafter referred to as “timepoint 3 years”.

At timepoint 3 years (between April and July of 2024), the study subjects provided venous blood samples and filled out questionnaires about their COVID-19 history (confirmed by laboratory testing—SARS-CoV-2 antigen or nucleic acid detection in the upper respiratory tract specimens), the severity of symptoms (mild/moderate/severe) as well as booster shot administrations.

All subjects provided informed consent for their participation in the study. Ethical approval of the study was obtained from the Bioethics Committee of Andrzej Frycz Modrzewski Krakow University, Krakow, Poland (protocol code KBKA/27/O/2024).

### 2.2. Laboratory Testing

The testing was performed on fresh blood sera. Anti-S SARS-CoV-2 IgG concentrations were measured with the LIAISON^®^ SARS-CoV-2 TrimericS IgG immunoassay run on the LIAISON^®^ XL analyzer (both DiaSorin S.p.A, Saluggia, Italy). The quantification of the antibodies is based on the intensity of the chemiluminescent signal. The results are expressed in binding antibody units (BAU/mL), and values over 33.8 BAU/mL are classified as positive. According to the LIAISON^®^ SARS-CoV-2 TrimericS IgG instructions for use (IFU), results higher than 520 BAU/mL correlate with a high neutralization capability in the microneutralization assay (≥1:80). The assay’s quantification range is between 4.81 and 2080 BAU/mL, which may be exceeded by sample dilution 1:20, according to the manufacturer’s instructions.

Anti-N SARS-CoV-2 Ab were assessed with the Abbott’s SARS-CoV-2 IgG immunoassay run on the Abbott Architect i2000sr analyzer (both Abbott, Sligo, Ireland). The results of this chemiluminescent microparticle immunoassay (CMIA) are expressed semi-quantitatively, as indices, calculated as a ratio of the measured chemiluminescence signals of sample and calibrator. Values over 1.4 are classified as positive.

### 2.3. Statistical Analysis

The STATISTICA software ver. 13.3 (TIBCO Software Inc., Palo Alto, CA, USA) was used, and the significance level was set at 0.05. The differences between antibody concentrations at different timepoints were analyzed with the Wilcoxon test. The Mann–Whitney U test was used to verify the statistical significance of the differences in antibody concentrations between two groups (sex and age subgroups, COVID-19 vs. no-COVID-19 subgroups). Comparisons between multiple subgroups were assessed with the Kruskal–Wallis test with the Bonferroni correction applied. Associations between age and COVID-19 were checked with Pearson’s chi-squared test of independence. R, Spearman’s coefficient of rank correlation was also used.

## 3. Results

### 3.1. Epidemiological and Clinical Data

The mean age of the study group (*n* = 99) three years after the first vaccination was 49 years (age range: 26–77 years), and there were 18 participants over 60 years old. The cohort consisted of 85 females and 14 males.

Analysis of the questionnaires revealed that there were 38 subjects claiming to never have had COVID-19. There were 41 subjects who had COVID-19 once, 14 people who had it two times, 5 individuals who had it three times, and 1 person reported suffering COVID-19 four times. A total of 22 subjects reported having COVID-19 over the last year of observation, confirmed by SARS-CoV-2 detection by antigen testing. The majority (16) of these COVID-19 cases were self-described as mild, 5 subjects suffered moderate symptoms, and only one vaccinee considered the symptoms severe.

The majority of the subjects (64 out of 99) received one booster dose of COVID-19 vaccine, and 26 participants had two booster shots. Out of the 99 people, 9 decided against additional vaccinations. However, only one participant decided on a booster over the last year of the observation. At timepoint 3 years, the median number of days since the last vaccine dose administration was 869 (ca. 2.38 years).

### 3.2. The Anti-SARS-CoV-2 Antibody Testing Results at Timepoint 3 Years

**Figure 1:**
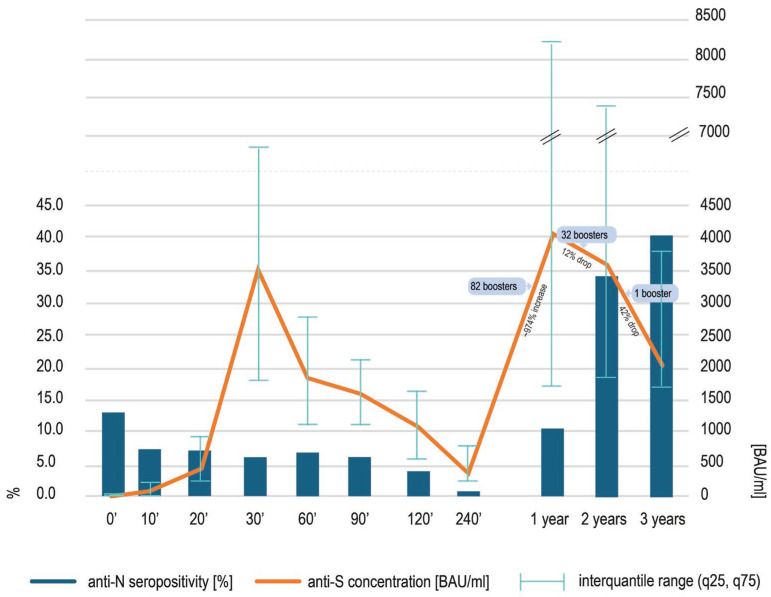
The concentrations of anti-spike SARS-CoV-2 IgG antibodies [orange line] at different timepoints after the first COVID-19 vaccination, presented as medians and interquantile range (q25, q75). The details of the previous findings—at timepoints 0′ to 2 years—may be found elsewhere [8,9,10,11]. At timepoint 3 years, a statistically significant (*p* < 0.0001, Wilcoxon test) decrease in anti-spike SARS-CoV-2 IgG was noted. Concomitantly, the percentage of participants with detectable anti-nucleocapsid antibodies [blue bars] increased, although this rise was not significant.

In comparison to the data obtained two years after the vaccination (3600 BAU/mL) [11], a statistically significant (*p* < 0.0001, Wilcoxon test) decrease in anti-spike SARS-CoV-2 IgG concentration was noted at three years, although the median concentration (2040 BAU/mL) was still high, close to the upper quantitation limit of the assay (2080 BAU/mL) (Figure 1). The lowest concentration noted was 414 BAU/mL, and the highest was 13,360 BAU/mL. Forty-six percent of the samples had to be diluted due to the results exceeding the upper limit of quantification of the assay. The decrease in the median anti-S antibody concentration seen over the third year of observation was more rapid than the decrease observed over the second year [11], probably due to the number of boosters decreasing from 32 [11] to 1. The concentration of anti-S SARS-CoV-2 IgG at three years in the only participant boosted over the preceding year was 1468 BAU/mL.

The number of anti-nucleocapsid positive individuals at timepoint 3 years was high and seropositivity reached 40.4% of the cohort, growing from 34% at timepoint 2 years (Figure 1). This rise was not statistically significant (*p* = 0.9467, Wilcoxon test).

### 3.3. Factors Influencing Anti-SARS-CoV-2 Antibody Titers at Timepoint 3 Years

The anti-spike SARS-CoV-2 IgG concentrations and anti-nucleocapsid SARS-CoV-2 titers at 3 years were analyzed in the context of epidemiological and clinical data provided in the questionnaires (Table 1).

We did not find statistically significant differences between the sexes in anti-S concentrations or in anti-N titers. The anti-S median concentrations as well as anti-N titers were statistically significantly higher in the older (above 60 y.o.) vs. younger (below 60 y.o.) subjects. The median anti-N titer in the older vaccinees was higher than the cut-off for a positive result (1.4).

The median anti-S concentration increased with the number of boosters accepted, but the differences were not statistically significant. The highest median titer of anti-N SARS-CoV-2 was noted for the participants who decided against booster vaccinations, which may show that they are more susceptible to infection, but the difference was also not significant.

There was no statistically significant dependence between the overall COVID-19 history or COVID-19 over the last year of observation and anti-S or anti-N antibody levels; however, the anti-S antibody concentration correlated inversely with the number of days from the last reported COVID-19 (Spearman rank correlation, R = −0.3218 and *p* = 0.0129).

**Figure 2:**
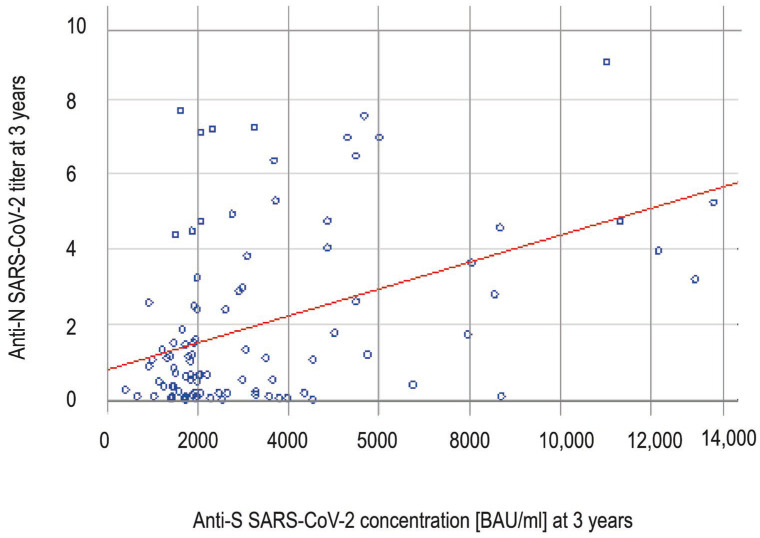
The correlation (Spearman rank correlation, R = 0.3574 and *p* = 0.0003) between anti-spike and anti-nucleocapsid SARS-CoV-2 antibodies at timepoint 3 years. Each blue dot represents the anti-N and anti-S results for a single participant. The anti-S concentration increases with rising anti-N titers.

At timepoint 3 years, the anti-S concentrations correlated positively with anti-N titers (Spearman rank correlation, R = 0.3574 and *p* = 0.0003) (Figure 2) and the level of anti-S antibodies was statistically significantly higher in the anti-N seropositive group than in the anti-N seronegative group (3160 BAU/mL in anti-N positive vs. 1932 BAU/mL in anti-N negative; *p* = 0.0001, Mann–Whitney U test).

### 3.4. The Relationship Between Anti-SARS-CoV-2 Antibodies and COVID-19 Incidence

As may be concluded from the above observations, anti-spike SARS-CoV-2 concentrations at timepoint 3 years seem to correlate better with anti-nucleocapsid titers than with the confirmed COVID-19. We attempted to verify this further by comparing anti-S median concentrations in the subgroups described below and in Table 2.

A total of 22 COVID-19 cases were confirmed by antigen testing [C+] over the last year of observation. Within this group, there were 10 anti-N positive COVID-19 convalescents [C+N+] and 12 anti-N negative COVID-19 cases [C+N−]. These subgroups differed in the median time between blood testing and COVID-19, which was shorter in the C+N+ subgroup (140 days, ca. 4.7 months vs. 211 days, ca. 7 months), although this time difference was not statistically significant (Student’s *t*-test, *p* = 0.2465). Additionally, in a further 20 cases, COVID-19 could be suspected based on anti-nucleocapsid SARS-CoV-2 results [C−N+] positive and increasing between timepoints 2 and 3 years. Overall, there were 42 cases of suspected SARS-CoV-2 infection.

The remaining 57 subjects did not report COVID-19, and their anti-N titers did not indicate infection [C−N−]. In 47 of these subjects, anti-nucleocapsid antibodies were not detected at timepoint 3 years, and 10 persons were seropositive at both 2 and 3 years but their anti-N titers decreased between these timepoints.

The median anti-S concentration in the subgroup of people who were suspected to have had COVID-19 was 2940 BAU/mL and was statistically significantly higher than in the participants with no hints of infection over the preceding year (1930 BAU/mL) (*p* = 0.0012, Mann–Whitney U test).

The highest anti-spike IgG concentration was noted in COVID-19 cases with detectable anti-nucleocapsid antibodies (subgroup C+N+; 6400 BAU/mL), followed by the subgroup with COVID-19 recognized solely based on anti-N testing results (C−N+; 3460 BAU/mL). The concentration observed in the subgroup of no suspected SARS-CoV-2 infection (C−N−; 1930 BAU/mL) was similar to the subgroup of COVID-19 convalescents with no detectable anti-N antibodies (C+N−; 1903 BAU/mL). Altogether, this data shows that anti-N seropositivity, most likely related to recent (over the previous 4–5 months) infection, is the stronger indicator of anti-SARS-CoV-2 immunity (presumed by higher anti-spike antibody concentrations) than the history of confirmed COVID-19.

## 4. Discussion

The quantitative anti-spike SARS-CoV-2 antibody immunoassays played an important role in the post-vaccination era of the COVID-19 pandemic. Testing people who had undergone vaccination proved the immunogenicity of the vaccines by demonstrating a skyrocketing concentration of protective antibodies, e.g., [12,13,14]. The antibody concentrations in vaccinated subjects were shown to be a few times higher than in COVID-19 convalescents, for example, van den Hoogen et al. [15] showed a median anti-spike SARS-CoV-2 IgG concentration in Comirnaty recipients of 2408 BAU/mL, in comparison to 91 BAU/mL in unvaccinated subjects.

The additional vaccine doses were recommended internationally [16], in response to the reports on the humoral immunity waning few months after vaccination, e.g., [17], and the concomitant appearance of SARS-CoV-2 variants [18,19]. The urge to maintain high anti-S antibody concentrations has been justified by data showing that the immunity against COVID-19 is correlated with the antibody titer [20,21]. Hollister et al. showed that each three-fold increase in anti-S RBD domain SARS-CoV-2 antibody titers reduced the odds of first-time post-vaccination infection by 21%, and higher anti-RBD S levels were associated with protection against reinfection with the Omicron variant [22].

Some attempts have been made to establish an antibody titer conferring immunity [23,24,25,26]. Similar approaches had been implemented in the past for other immunizations, e.g., against the rabies virus, where results of virus neutralization tests were shown to correlate with binding antibody titers measured with ELISA, and a minimal concentration of 0.5 IU/mL was considered protective [27]. As for COVID-19 immunity, Feng et al. correlated 90% protection against a symptomatic SARS-CoV-2 infection over the following 4–6 months with an anti-spike SARS-CoV-2 IgG concentration of 899 BAU/mL [28]. However, no universal protective concentration has been established thus far.

This study demonstrated that 3 years after the primary COVID-19 mRNA vaccination, the median anti-spike (S) SARS-CoV-2 antibody concentration declined, dropping from 3600 BAU/mL at 2 years to 2040 BAU/mL. This may have been related to a drastic decrease in booster dose acceptance in our cohort. Only 1 out of 99 participants of this study decided to accept additional dose of the COVID-19 vaccine over the third year after the primary vaccination and the median time from the last vaccination in our cohort was 2.38 years. The need for boosters has been underscored by research, see, e.g., [29], but it was also emphasized that natural infections shape cross-immunity against SARS-CoV-2 variants [30] and hence are an important component of the hybrid immunity—built on the foundation of vaccination and boosted with even asymptomatic infections [31,32,33].

Without boosters (only 1 in 99 subjects), the median concentration of anti-S antibodies declined over the third year of our study, and this drop was more pronounced than the one observed over the preceding year (12% vs. 42%), when 32 boosters had been delivered [11]. The lower level of anti-S antibodies could have been the reason for more COVID-19 cases, suspected on the basis of an increasing anti-nucleocapsid (N) seropositivity. On the other hand, these SARS-CoV-2 infections, both recognized and paucisymptomatic or asymptomatic, might have contributed to a sustainably high anti-S median concentration (2040 BAU/mL) at timepoint 3 years. Indeed, the vaccinees with COVID-19 suspected over the last year of observation presented with statistically significantly higher anti-spike antibody concentrations and anti-S SARS-CoV-2 concentrations correlated positively with anti-nucleocapsid SARS-CoV-2 titers. As previously seen in [11], when boosters were shown to be the main inducers of immunity, we did not see the correlation between the anti-S and anti-N antibodies. Currently, in populations practically not boosted, a natural contact with SARS-CoV-2 seems to be the crucial stimulus of immunity.

Anti-nucleocapsid antibodies (indicating infection in COVID-19 mRNA vaccine recipients) are not always found in COVID-19 convalescents, and vice-versa: anti-N IgG may be detected in individuals without known COVID-19 history. Therefore, in our study we decided to analyze the cases of suspected COVID-19 after the consideration of both the history of positive SARS-CoV-2 antigen or RNA testing results (confirmed cases) and anti-N serology results. This resulted in 42 suspected COVID-19 cases over the last year of observation of our cohort. At timepoint 3 years after vaccination, anti-nucleocapsid antibodies were detected in as much as 40% of participants, whereas the reported COVID-19 incidence, confirmed by SARS-CoV-2 antigen or RNA detection in the upper respiratory tract specimens, dropped from around 39% at 2 years to 22.22% at 3 years. This discrepancy may be explained by the decreasing number of reported cases in the general population and the decreasing volume of testing for COVID-19 in Poland. The former is reflected by data gathered by the Polish National Institute of Public Health—National Institute of Hygiene [34]—which shows 511 555 COVID-19 cases between the third quarter of 2022 and second quarter of 2023 (respective to the second year of our observation), in comparison to 231 446 cases between the third quarter of 2023 and second quarter of 2024 (respective to the third year of our observation). As for the latter, the volume of testing for COVID-19 (SARS-CoV-2 nucleic acid and/or antigen) in our Poland-wide laboratory network decreased substantially in the respective periods—from around 300 000 to around 5 000 tests performed. This could have been the outcome of the lower virulence of the circulating SARS-CoV-2 variants, leading to milder symptoms and fewer people seeking doctors’ advice or COVID-19 testing. Similarly, Premkumar et al. observed 60.1% of anti-N seropositivity versus 23.7% reported COVID-19 in their healthcare workers’ cohort, suggesting a high rate of unrecognized SARS-CoV-2 infections [35].

On the other hand, anti-nucleocapsid antibodies are not always found in COVID-19 convalescents, which may be attributed to the quick waning of these antibodies in blood. In our cohort, 12 out of 22 confirmed COVID-19 cases were negative for anti-N IgGs at timepoint 3 years. We analyzed the median time between the blood testing and confirmed COVID-19 and noted that it was shorter in anti-N positive (140 days; ca. 4.7 months) in comparison to anti-N negative (211 days; ca. 7 months) individuals. This is in line with the observations made at the beginning of the pandemic, when it was established that anti-nucleocapsid antibodies are typically detected for only a few months after the infection [36], although they may last longer following a more severe disease course [37,38].

It might seem concerning that the high anti-spike antibody levels in our vaccinees did not prevent SARS-CoV-2 infections. The number of suspected COVID-19 cases over the last year of the study was high (42 out of 99 subjects), no matter that the median antibody concentration measured at the preceding timepoint of 2 years (3600 BAU/mL) exceeded the proposed protective titers (e.g., 899 BAU/mL [28]). However, 20 of the suspected cases had not even been aware of having COVID-19, and out of 22 subjects with confirmed COVID-19 between 2 and 3 years after the primary vaccination, only 1 reported the symptoms as severe. This shows that COVID-19 vaccinations prove effective in their primary goal—preventing severe symptoms and/or hospitalization.

There are some limitations of our study. First, we only investigated the immunity conferred by the presence of antibodies in blood. It must be noted that this is only a part of the multifaceted SARS-CoV-2 protection, and an important role is also played by cellular response and memory cells [39]. Further, there is an overrepresentation of females in our cohort. It is known that sex influences the functions of the immune system and women tend to have greater humoral immunity [40]. However, over the course of our study, the relationship between anti-S SARS-CoV-2 IgG concentration and sex lost significance [10,11], which might mean that over longer periods, the influence of sex becomes negligible, and other factors impact the antibody results more strongly, though this would require further studies on larger cohorts. It may also be argued that the results of a study on healthcare workers may be misleading when projected onto the general population. The exposure of HCW to COVID-19 cases may be increased and their awareness may skew their choices towards protective measures. However, as shown, e.g., in [41], HCWs’ adherence to precautionary guidelines decreased after being vaccinated, and thus this bias may be less pronounced.

## 5. Conclusions

Our study shows that in individuals vaccinated against COVID-19, a high concentration of anti-spike SARS-CoV-2 IgGs persists for at least 3 years. This is accompanied by an increase in anti-nucleocapsid seropositivity, which indicates a substantial rate of contact with SARS-CoV-2. However, the infections tend to be asymptomatic (recognized solely by seroconversion in anti-N antibodies) or reported as mild.

## Figures and Tables

**Table 1:** Median anti-spike and anti-nucleocapsid SARS-CoV-2 IgG in different subgroups at 3 years after the first vaccination.

*n* = 99		*n*	Anti-Spike SARS-CoV-2 IgG Median Concentration [BAU/mL]		Anti-Nucleocapsid SARS-CoV-2 IgG Median Titer	
Sex	Female	85	2010	*p* = 0.6769 Mann–Whitney U	1.06	*p* = 0.5369 Mann–Whitney U
	Male	14	2440		0.87	
Age	Below 60 y.o.	81	1990	*p* = 0.0083 Mann–Whitney U	0.71	*p* =0.0242 Mann–Whitney U
	Over 60 y.o.	18	3360		3.44	
Number of vaccine boosters	0	9	1898	*p* = 0.7679 Kruskal–Wallis	1.53	*p* = 0.6989 Kruskal–Wallis
	1	64	2045		0.79	
	2	26	2145		1.05	
COVID-19 history	No	38	2055	*p*= 0.5080 Mann–Whitney U	1.12	*p* = 0.6607 Mann–Whitney U
	Yes	61	2005		0.89	
COVID-19 over the last year	No	77	2000	*p* = 0.2720 Mann–Whitney U	1.03	*p* = 0.3634 Mann–Whitney U
	Yes	22	2620		1.20	

**Table 2:** Median anti-spike SARS-CoV-2 IgG concentrations 3 years after the primary vaccination in subgroups divided on the basis of suspected COVID-19.

*n* = 99		Anti-S SARS-CoV-2 Concentration at Timepoint 3 Years [BAU/mL]	Statistical Significance Between Subgroups CN						
COVID-19 Status Between 2 and 3 Years Since the First Vaccination:									
	***n*** **:**		**C+N+**	**C−N+**		**C+N−**			**C−N−**
COVID-19 reported, confirmed by anti-N [C+N+]	10	6400		1.0000		0.0156 **			0.0030 **
COVID-19 reported, not confirmed by anti-N [C+N−]	12	1903	0.0156 **	0.0596					1.0000
COVID-19 not reported, but indicated by anti-N [C−N+]	20	3460	1.0000			0.0596			0.0068 **
Altogether suspected COVID-19	42	2940 *							
No suspected contact with SARS-CoV-2 [C−N−]	57	1930 *	0.0030 **		0.0068 **		1.0000		

‘C’ denotes reported, antigen testing verified COVID-19 status; ‘N’ denotes anti-nucleocapsid status. * Participants with suspected COVID-19 (reported [C+] and/or indicated by anti-N [N+] antibodies) demonstrated statistically significantly higher anti-spike SARS-CoV-2 antibodies than subjects with no hints of SARS-CoV-2 infection (*p* = 0.0012, Mann–Whitney U test). ** The statistical significance of the differences in the anti-spike concentrations between subgroups C+N+, C−N+, C+N−, and C−N−, analyzed with the Kruskal–Wallis test.

## Data Availability

The data presented in this study are available on reasonable request from the corresponding author.

## Abbreviations

The following abbreviations are used in this manuscript:

Ab	Antibody
BAU/mL	Binding Antibody Units
COVID-19	Coronavirus Disease 2019
HCW	Healthcare Workers
N	Nucleocapsid
SARS-CoV-2	Severe Acute Respiratory Syndrome Coronavirus 2
S	Spike
